# Ecosystem services in European protected areas: Ambiguity in the views of scientists and managers?

**DOI:** 10.1371/journal.pone.0187143

**Published:** 2017-11-15

**Authors:** Christiaan Hummel, Antonello Provenzale, Jaap van der Meer, Sander Wijnhoven, Arno Nolte, Dimitris Poursanidis, Guyonne Janss, Matthias Jurek, Magnus Andresen, Brigitte Poulin, Johannes Kobler, Carl Beierkuhnlein, João Honrado, Arturas Razinkovas, Ana Stritih, Tessa Bargmann, Alex Ziemba, Francisco Bonet-García, Mihai Cristian Adamescu, Gerard Janssen, Herman Hummel

**Affiliations:** 1 Department of Estuarine and Delta Systems, NIOZ Royal Netherlands Institute for Sea Research and Utrecht University, Yerseke, the Netherlands; 2 VU University, Amsterdam, the Netherlands; 3 Consiglio Nazionale delle Ricerche, Rome, Italy; 4 Department of Coastal Systems, NIOZ Royal Netherlands Institute for Sea Research and Utrecht University, Texel, the Netherlands; 5 EcoAuthor, Heinkenszand, the Netherlands; 6 Deltares, Delft, the Netherlands; 7 Foundation for Research and Technology, Crete, Greece; 8 Estación Biológica de Doñana, CSIC, Almonte, Spain; 9 United Nations Environment, Vienna, Austria; 10 Research Institute for the Conservation of Mediterranean Wetlands, Tour du Valat, Le Sambuc, France; 11 Umweltbundesamt, Vienna, Austria; 12 BayCEER, University of Bayreuth, Bayreuth, Germany; 13 InBIO/CIBIO, Faculdade de Ciências, Universidade do Porto, Porto, Portugal; 14 Marine Science and Technology Centre, Klaipeda University, Klaipeda, Lithuania; 15 Eidgenössische Technische Hochschule, Zürich, Switzerland; 16 University of Bergen, Bergen, Norway; 17 University of Granada, Granada, Spain; 18 University of Bucharest, Bucharest, Romania; 19 Rijkswaterstaat, Leeuwarden, the Netherlands; Universita degli Studi di Genova, ITALY

## Abstract

Protected Areas are a key component of nature conservation. They can play an important role in counterbalancing the impacts of ecosystem degradation. For an optimal protection of a Protected Area it is essential to account for the variables underlying the major Ecosystem Services an area delivers, and the threats upon them. Here we show that the perception of these important variables differs markedly between scientists and managers of Protected Areas in mountains and transitional waters. Scientists emphasise variables of abiotic and biotic nature, whereas managers highlight socio-economic, cultural and anthropogenic variables. This indicates fundamental differences in perception. To be able to better protect an area it would be advisable to bring the perception of scientists and managers closer together. Intensified and harmonised communication across disciplinary and professional boundaries will be needed to implement and improve Ecosystem Service oriented management strategies in current and future Protected Areas.

## Introduction

Marine and terrestrial ecosystems play a vital and ever increasingly important role in providing essential Ecosystem Services to humanity and society [1]. Ecosystem Services (ES) are the benefits that humans derive from ecosystems, ranging from material benefits such as food or fuel, to non-material benefits such as soil formation, water purification, recreation or aesthetics [1–3]. Due to their societal relevance and their close link to the state of ecosystems and the broader environment [4], ES have increasingly been used as an assessment and policy communication tool [5–7].

Over the course of the last century strong anthropogenic pressures have caused widespread habitat degradation and a noticeable decline in the environmental quality across many ecosystems, potentially leading to biodiversity loss and an increased risk of declining or even collapsing ecosystem functions, and subsequent loss of ES [1, 8–13].

The very first Protected Areas (PAs) in the form as we now know them can be traced back to the nineteenth century. The first ‘modern’ protected area was Yellowstone National Park, founded in 1872, as “a public park or pleasuring ground for the benefit and enjoyment of the people” [14]. Around the globe similar types of protected areas have been set up ever since. The reasons to protect the environment through PAs however were of a different nature in several regions around the globe. In North America they were set up to protect dramatic and sublime scenery, in Africa to protect game and their habitats in order to maintain elite hunting traditions [15, 16], and in Europe to protect the landscape [17]. This means that already from the beginning PAs were installed to protect specific ES and (bio)diversity, although the aims of these PA were not meant specifically to protect ES or (bio)diversity. The focus in using the terms biodiversity and ES with regard to the management of PA arose only in the eighties and nineties of the last century with the Convention on Biological Diversity [18] and the onset of ES studies [19, 20].

Nowadays, Protected Areas have become a key component of nature conservation, human well-being and also of management and policy strategies from regional to global scales [21–23]. They can play an important role in counterbalancing the impacts of ecosystem degradation [24], avoiding collapse of ecosystem function, and also mitigating the associated loss of ES, not only inside but also outside the PA [25–30]. The European network of PAs can make a substantial contribution to fulfil the requirements of various conventions and directives, including the Convention on Biological Diversity (CBD) through maintaining the natural heritage of European ecosystems. This is supported by the diversity and the spatial distribution of PAs across the whole continent. However, direct and indirect human pressures on biodiversity such as climate and land use change have wide reaching impacts [31] especially affecting mountains and transitional coastal ecosystems, which are particularly sensitive to environmental changes [32]. Therefore, for an optimal protection of a PA and a better environmental quality, thereby strengthening a sustainable delivery of current services and for the future, it is essential to account for the pressures that may pose major threats to the system [33, 34].

In the pursuit of identifying the most important variables in European PAs, the EcoPotential project (http://www.ecopotential-project.eu) surveyed the state-of-art view on the services and pressures in a representative selection of areas covering a variety of European regions. This survey elicited responses from environmental scientists as well as PA managers, and for two main groups of PAs, mountainous and transitional waters. In the surveys the importance of various biotic, abiotic, and socio-economic variables for the ecosystem services and pressures in different PAs were assessed.

A mismatch between academic and management perceptions of ecosystem services and management priorities may well result in important shortcomings for the application of research outputs in adaptive PA management. To tackle this issue, here we will assess the similarities and differences in the vision of environmental scientists versus PA managers on which ecosystems services and pressures are most important in their PA. We also assess whether these variables identified by scientists and managers are of biotic, abiotic or socio-economic/anthropogenic nature. As the respondents’ perception of these variables was the central topic of the assessment, the definition of importance was left open to their interpretation. In general, we hypothesised that there would be differences in perceptions between scientists and managers due to their daily work routine, and between mountainous PAs and transitional water PAs.

## Material and methods

The importance of various variables underlying the ecosystem services and threats in transitional waters (marine coastal waters, deltas, lagoons) and mountainous PAs were assessed in two surveys; one survey distributed among environmental scientists (hereafter called ‘scientists’) and the other distributed among the managers of the studied PAs. The link with the ecosystem structures and functions of these areas was only assessed in the survey distributed among the scientists. The surveys were sent by email to 15 scientists working in the EcoPotential project, and 11 managers of protected areas were interviewed face to face by scientists working in the EcoPotential project.

**Fig 1:**
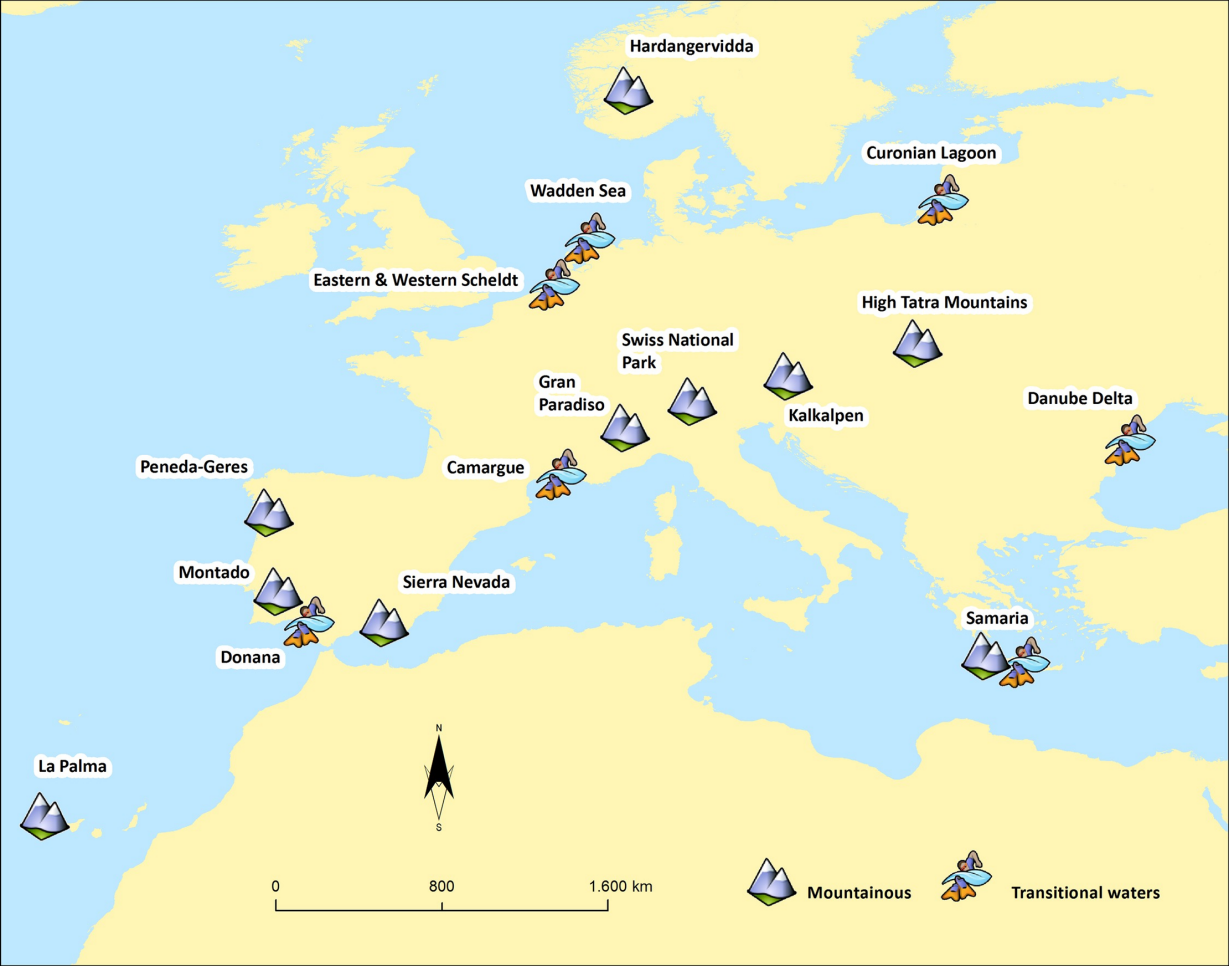
Overview of PAs surveyed in Europe. Mountain symbol = mountainous PA, wave symbol = transitional waters PA (figure is for illustrative purposes only).

To be able to obtain a proper overview of the major variables important for environmental scientists and PA managers in Europe, a broad range of PAs with different biogeographic settings and environmental conditions were included in the analyses (Fig 1). The analyses included transitional waters, such as the Wadden Sea in the Netherlands, the Curonian Lagoon in Lithuania, the Danube Delta in Romania, and the Camargue in France, as well mountainous areas, such as the Gran Paradiso in Italy, the Nördliche Kalkalpen in Austria, the Sierra Nevada in Spain, and Peneda-Gerês in Portugal (Table 1). All of these areas are recognised PAs having one or more of the following designations: National Park status, Natura 2000, UNESCO World Heritage area, or UNESCO Biosphere Reserve (Table 1).

**Table 1:** Protected areas surveyed in the study including country and protection status.

		*Transitional Waters*		*Mountains*		
	*Country*	*Scientists*	*Managers*	*Scientists*	*Managers*	*Protection status*
*Camargue*	F	**+**	**+**			UBR, N2
*Curonian Lagoon*	LT	**+**	**+**			NP, N2,UWH
*Danube Delta*	RO	**+**				N2, UBR, UWH
*Doñana*	E	**+**	**+**			NP, N2, UBR, UWH
*Eastern Scheldt*	NL	**+**				NP, N2
*Wadden Sea*	NL	**+**	**+**			NP, N2, UBR, UWH
*Western Scheldt and Saeftinghe*	NL	**+**				N2
*Samaria*	GR	**+**	**+**	**+**	**+**	NP, N2, UBR
*Gran Paradiso*	I			**+**	**+**	NP, N2,
*Hardangervidda*	N			**+**	**+**	NP
*High Tatra Mountains*	SK			**+**	**+**	NP, N2, UBR
*La Palma*	E				**+**	NP, N2, UBR
*Kalkalpen National Park*	A			**+**	**+**	NP,N2
*Oros Idi*	GR			**+**		NP, N2
*Peneda-Gerês*	P			**+**		NP, N2, UBR
*Sierra Nevada*	E			**+**		NP, N2, UBR
*Swiss National Park*	CH				**+**	NP, UBR

NP: National Park, UBR: Unesco Biosphere Reserve, N2: Natura 2000 site, UWH: Unesco World Heritage

In the survey for scientists they were asked to identify the major ecosystem types for the PA and the most important ecosystem services in these ecosystems (for all ecosystem types encountered see S4 Table, for an example of the survey see S1 Table). Subsequently the major ecosystem functions and structures underlying the most important services had to be indicated, and lastly the major threats to these ecosystem services, functions and structures.

The relative number of times a variable was mentioned in a category (ecosystem services or threats) per PA, across all ecosystem types, was adopted as the degree of importance of that variable in a given PA. The importance of each variable was then averaged over all surveyed PAs, and the standard error was calculated. Mean importance values of less than 2% were not included in further analyses.

To overcome the critical issue that often similar variables were assigned by scientists with several different names, the variables were harmonised to a standard set of variables. An overview of this harmonisation of variables is given in S2 Table. After harmonisation, all variables were categorised in variables of biotic, abiotic and socio economic nature for ES, and of biotic, abiotic and anthropogenic nature for threats (details can be found in S2 Table). The categorisation of the variables is dependent on the origin of the variable, to prevent loss of causality. For example: the ES aquaculture is categorised as biotic since the object in aquaculture is of biotic origin, and the ES materials of economic use as abiotic since the materials are of abiotic origin, though both could be considered to be socio-economic, because both are an economic activity. If both would have been categorised as socio-economic, the origin of the variable (abiotic or biotic) would be lost, and with this the possible connections and implications for the supporting (functions in the) (eco)system.

To remain as close as possible to the original answers given by managers and scientists we have chosen for the analyses not to use the existing ES classification schemes of the Millennium Ecosystem Assessment [1], TEEB [35] and CICES [36], also because they lack an integrated approach for classifying the EF and threats, making it hard to harmonise all variables in the same way. Moreover, using the original variables as given by managers and scientists as much as possible makes it easier to distinguish between the different answers and different views of scientists and managers.

Some variables were miscategorised by the scientists. For example “water supply” was indicated as an ecosystem function whereas it is an ecosystem service. For further analysis, and to overcome this type of flaw, the variables were matched with the contextually most similar variable within a category. In this specific case “water supply” was matched with the variable “hydrodynamics” in the category of Ecosystem functions and structures (all incorrectly categorised variables are summarised in S5 Table; the “corrected” variables are included in S2 Table).

During the survey, PA managers were asked to indicate the major ecosystem services and threats in their protected area (for an example of the survey see S3 Table). Next, they were asked to indicate what the relative importance of each service and threat was. For services we have used the standard 5 point Likert scale [37] (0 = not present, 1 = very low importance, 2 = low importance, 3 = moderate importance, 4 = high importance, 5 = very high importance). For threats we have adopted the 3 point IPCC scaling for Risks [38] (0 = no threat, 1 = low to moderate threat, 2 = strong threat, 3 = very strong threat). The counts of importance for each variable were averaged over all surveyed PAs, indexed (max score is 100%), and the standard error was calculated.

In each survey the total importance of all variables mentioned by a scientist or a manager for each category (i.e. the ES and threats) in each PA always summed up to 100%. The (average) relative importance of the specific variables, as viewed by all scientists and PA managers, both within and between the two different types of PAs, i.e. Transitional Waters and Mountains, were compared after examining for normality using a Kolmogorov-Smirnov test, and statistically analysed for significant differences by means of a Mann Whitney U Test [39].

All underlying data and analyses will be made available at publication through open access at https://doi.org/10.6084/m9.figshare.5513530.v1

## Results

### Ecosystem services

**Fig 2:**
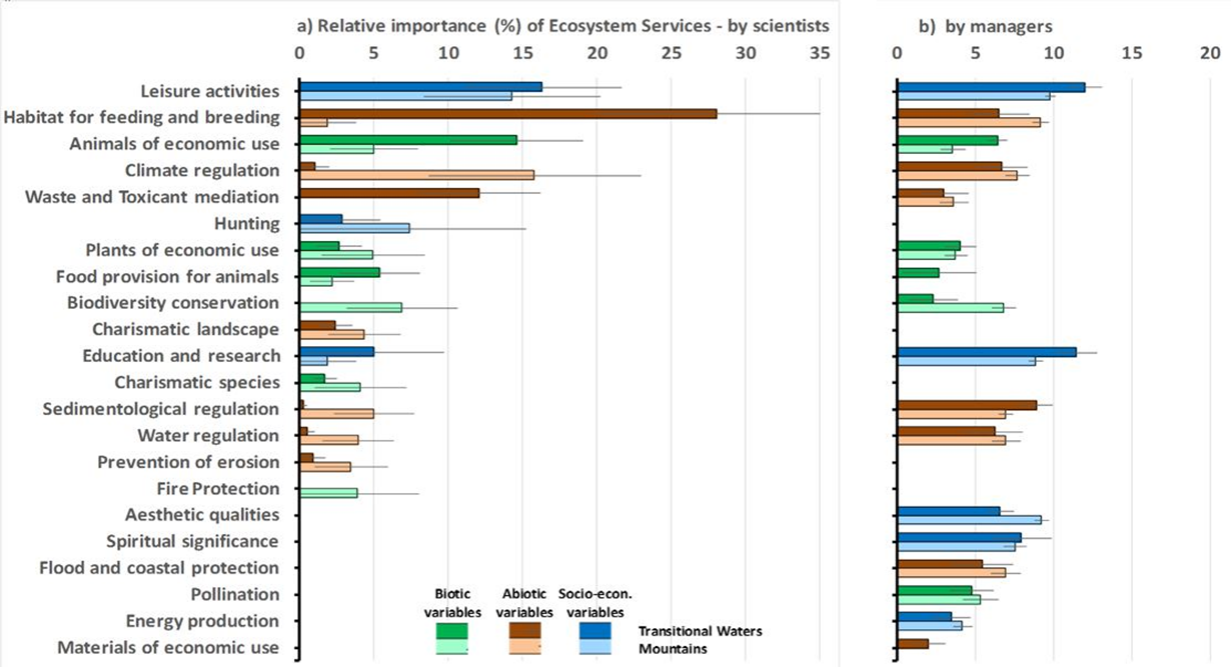
Relative importance (in %) of ecosystem services by scientists and PA managers in Transitional Waters and Mountains. (a) indicates scientists (b) indicates PA managers. Upper row (darker colours) indicates Transitional Waters, lower row (lighter colours) indicates Mountains, separated in ecosystem services of biotic (green), abiotic (brown) and socio-economic (blue) nature (indicated are averages and standard errors).

The 5 most important ES for scientists were: leisure activities, habitat for feeding and breeding, animals of economic use, climate regulation, and waste and toxicant mediation (Fig 2A). The scientists of transitional waters and those of mountainous PAs often had a strongly, sometimes significantly, different view on the level of importance of these ES (Table 2). For example, scientists of transitional waters indicated habitat for feeding and breeding as very important, whereas for scientists of mountainous areas the habitat was hardly important but climate regulation was much more important (Fig 2A).

**Table 2:** Statistical significance of the difference in importance to scientists (SC) and Managers (MA) of ecosystem services and Threats for Mountainous (MO) and Transitional Water (TW) Protected Areas (SC+MA means the data of SC and MA pooled together; MO+TW means the data of MO and TW pooled together). –indicates no significant difference, ● means significant difference at the level p ≤ 0.05, ●● means p ≤ 0.01, and ●●● means p ≤ 0.005.

	MO vs. TW			SC vs. MA			
	*SC+MA*	*SC*	*MA*	*MO+TW*	*MO*	*TW*	
***Ecosystem Services***							
Leisure activities	-	-	-	-	-	-	
Habitat for feeding and breeding	-	●	-	-	●	-	
Animals of economic use	-	-	-	-	-	-	
Climate regulation	●●	●	-	-	-	●●	
Waste and Toxicant mediation	●	●	-	-	●	-	
Hunting	-	-	-	-	-	-	
Plants of economic use	-	-	-	●	-	-	
Food provision for animals	-	-	-	-	-	-	
Biodiversity conservation	●●	-	-	●	-	-	
Charismatic landscape	-	-	-	●●	-	●	
Education and research	-	-	-	●●●	●●	●	
Charismatic species	-	-	-	●	-	-	
Sedimentological regulation	-	-	-	●	-	●●●	
Water regulation	-	-	-	●	-	●●	
Prevention of erosion	-	-	-	-	-	-	
Fire Protection	-	-	-	-	-	-	
Aesthetic qualities	-	-	●	●●●	●●●	●●●	
Spiritual significance	-	-	-	●●●	●●●	●●●	
Flood and coastal protection	-	-	-	●	●	-	
Pollination	-	-	-	●	-	●●●	
Energy production	-	-	-	●●●	●	●●●	
Materials of economic use	●●	-	-	-	-	-	
Total number significant differences	**4** / 22	**3** / 22	**1** / 22	**12** / 22	**7** / 22	**9** / 22	
***Threats***							
Climate change	-	-	-	-	-	-
Overexploitation	-	-	-	-	-	●
Fire	-	-	-	●	●	-
Habitat loss	-	-	-	-	-	-
(Illegal) human activities	-	-	-	-	-	-
Exotic species	-	-	-	●●●	-	●
Pollution	-	-	-	-	-	-
Disturbance	-	-	-	●●	●	-
Hydrological changes	-	-	-	-	-	●
Change in species	-	-	-	●	-	●
Change in land use	-	-	-	●	-	-
Encroachment	-	-	-	-	-	-
Hydrological changes	●●●	●●●	-	●	-	-
Diseases	-	-	-	-	-	-
Tourism	-	-	-	●●●	●●	●●●
Eutrophication	-	●	-	●●●	●●	●
Predation	-	-	-	-	-	-
Landscape disturbance	-	-	-	-	-	-
Agriculture	-	-	-	●●●	●●	●●●
Fisheries	●	-	●●	●●●	-	-
Total number significant differences	**2** / 20	**2** / 20	**1** / 20	**10** / 20	**5** / 20	**7** / 20

PA managers also considered leisure activities and habitat for feeding and breeding to be important ES (Fig 2B), although the importance of habitat was lower than with scientists (in mountainous areas even significantly less important; Table 2). Among the 5 most important ES identified by managers were education and research, sedimentological regulation, and aesthetic qualities, which were all judged by scientists to be of significantly less importance (Fig 2B; Table 2).

Moreover, among PA managers, the difference in importance of most ES between transitional water and mountainous PAs was much smaller than among scientists (Fig 2; see also Table 2 second versus third column).

It became clear that scientists put more emphasis on the biotic and abiotic (system related) ES, whereas PA managers put more emphasis on the socio-economic and cultural ES (Fig 2).

### Pressures and threats

**Fig 3:**
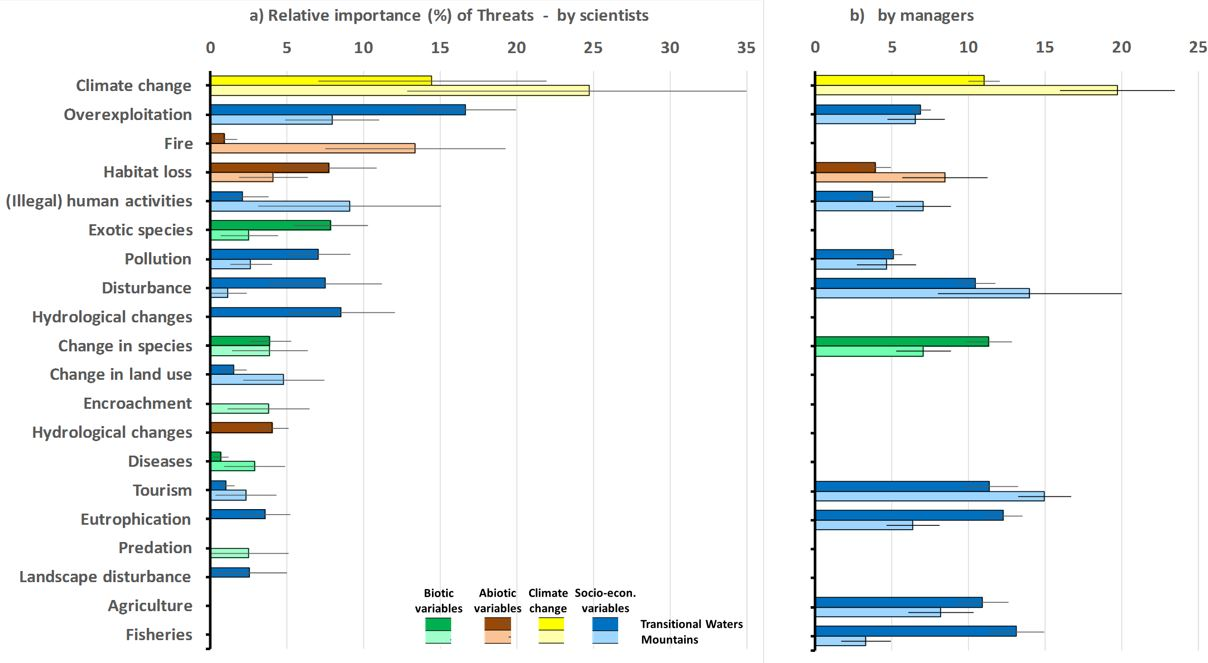
Relative importance of threats by scientists and PA managers in Transitional Waters and Mountains. (a) indicates scientists (b) indicates PA managers. Upper row (darker colours) indicates Transitional Waters, lower row (lighter colours) indicates Mountains, separated in biotic (green), abiotic (brown), climate change (yellow), and anthropogenic (blue) threats (indicated are averages and standard errors).

The most important threat to ecosystem services and underlying functions according to both scientists and PA managers was climate change (Fig 3).

Furthermore, for scientists the overall top 5 also contains two abiotic and two anthropogenic threats (Fig 3A), overexploitation and habitat loss, which were more important for transitional waters, while fire and illegal activities were more important for mountainous areas.

**Fig 4:**
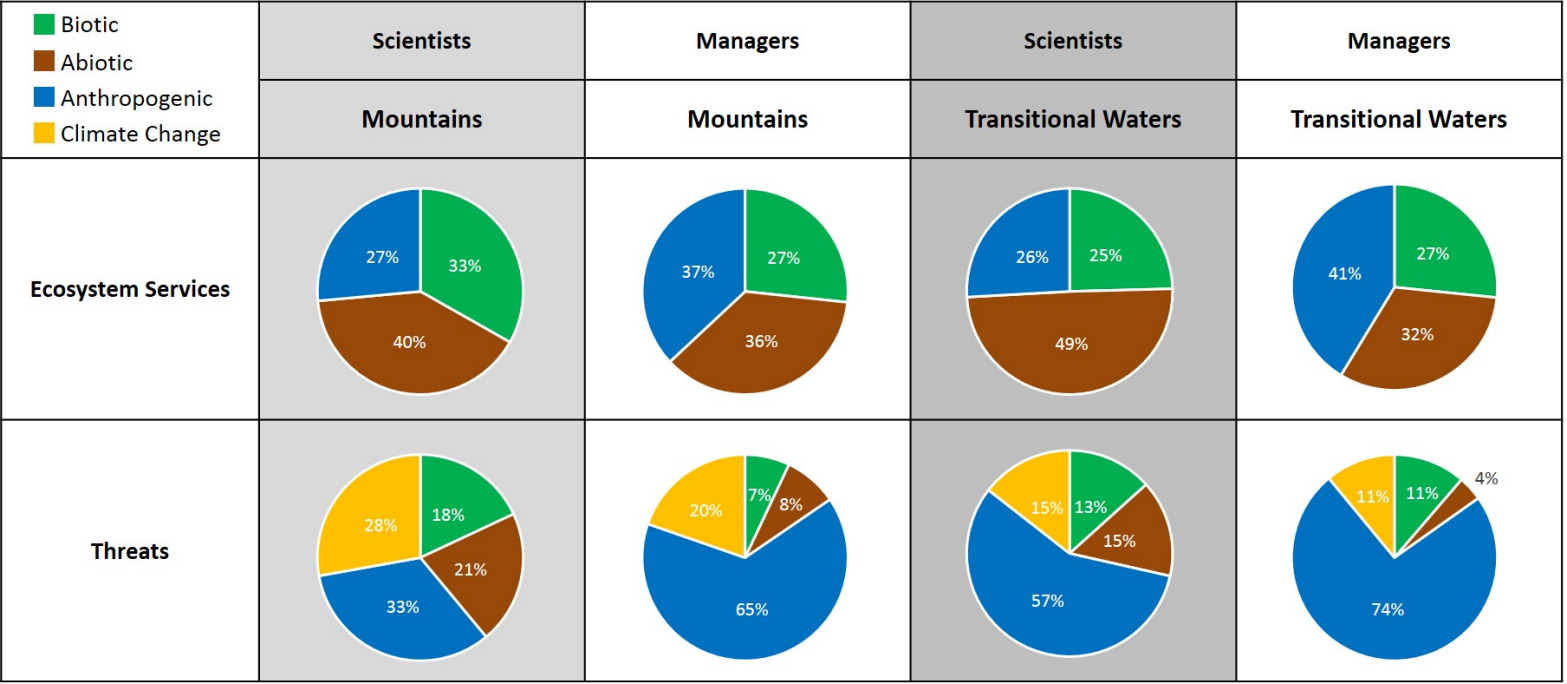
Overall importance of ecosystem services and threats for both scientists and PA managers in Transitional Waters and Mountains. Importance is separated in biotic (green), abiotic (brown), climate change (yellow), and anthropogenic (blue) variables.

For PA managers the most important threats besides climate change consisted solely of anthropogenic pressures (Fig 3B). PA managers hardly name any abiotic or biotic threats (see also Fig 4).

For threats the same holds as for ES, among PA managers the difference in importance of most threats between transitional water and mountainous PAs was much smaller than among scientists (Fig 3).

### Biotic, abiotic and anthropogenic variables

Summing up the variables and distinguishing between those of biotic, abiotic and anthropogenic (or socio-economic and cultural) nature showed differences in perception by scientists and managers, and differences between mountainous areas and transitional waters. This shows that the importance of biotic ES was considered higher among scientists of mountainous PAs than in transitional waters (Fig 4), whereas the abiotic ES were more important in transitional waters. PA managers indicated ES of anthropogenic nature as most important for both transitional waters and mountainous areas (Fig 4).

Regarding threats, PA managers indicated those of anthropogenic origin to be by far the most important, and threats of biotic and abiotic nature were least important (third row, Fig 4). Scientists also indicated a high importance of anthropogenic threats, but also a considerable importance of biotic and abiotic threats. In mountainous areas climate change was considered a major threat by scientists, while it was considered less of a threat for transitional waters.

### Variability of perception

An analysis of variance showed a remarkable difference in the perception of the importance of variables between scientists and PA managers. The variation in perception of important ecosystem functions, services, and threats in mountainous and transitional water PAs was threefold higher among scientists than among PA managers (Table 3; also compare error bars of Fig 2A with 2B and Fig 3A with 3B).

**Table 3:** Coefficient of variation (CV) in the relative importance of ecosystem services (ES) and threats (Thr) indicated by scientists and PA managers, for transitional water PA (TW) and for mountainous PA (MO).

*Domain*	*Variable*	*CV among Scientists*	*CV among PA Managers*
*TW*	ES	1.15	0.55
*TW*	Thr	1.25	0.30
*MO*	ES	1.82	0.28
*MO*	Thr	1.63	0.72
*Average*		**1.46**	**0.46**

Irrespective of the large variability in the perception of the importance of ES and threats by the scientists, a strong significant difference occurred in the level of importance for most (two-thirds) of the ES and threats as indicated by scientists versus those indicated by managers (Table 2).

When comparing the perception of the importance of ES and threats in mountainous PAs with those in transitional water PAs, the differences between both types of PAs were mostly non-significant in the view of scientists as well as in the view of managers (Table 2).

## Discussion

The results show that common categories of ES and threats are considered to be important across transitional water as well as mountainous PAs. This would allow to make a harmonised list of most important variables of ES and threats over both geographic domains. Such a harmonised list may in the future be helpful to overcome the difference in vocabulary between scientists and managers. This may also help include ES in PA management, since until now PA managers expressed that they did not explicitly apply the ES approach in their management, with only a few exceptions [40, 41].

A noteworthy result of this study is that the variables mentioned, and the importance given to these variables by scientists and managers, are dissimilar. The overall view on important ES and threats by scientists does not match the view of PA managers. Although the set-up of the surveys (offering scientists a blank page, and managers a list that indicated potential variables) may have enhanced the differences, both groups had the liberty to identify variables of their choice which they regarded to be important. Moreover, the differentiation is also very apparent at a higher organisational level of factors. Scientists gave more importance to variables of abiotic and biotic nature, whereas the PA manager’s view was that the socio-economic, cultural and anthropogenic variables are more important. This indicates that there are fundamental differences in the perception of various categories of variables.

In relation to this with regard to threats, it has been found that managers may have a low perception of environmental risks, which may explain a lower variability in views, yet at the same time may be reason for incidental strong mismatches between managers [42].

A potential reason managers emphasise anthropogenic ES and threats more than scientists may be related to the fact that managers deal with various stakeholders, like municipalities, local businesses, farmers and fishermen, in day to day management of a PA [43, 44]. Thereby, they bring aspects such as disturbance, tourism and agriculture more to the foreground, since these are the elements they are faced with on a more regular basis. Scientists on the other hand, have less interaction with stakeholders, and seemingly focus more on the functional aspects underlying the services [45], and thereby regard these functional aspects to be of more or equal importance.

An important difference in the perception of the system by scientists and managers may also be caused by the spatial and temporal frame in which they observe the system. While scientists often model and observe long-term, large-scale processes and changes, managers commonly deal with decision-making on annual or sub-annual timeframes, and at local scales (for example managing tourist numbers or issuing licenses). Because of this, scientists are likely to pay more attention to long term processes, while managers will give more weight to issues they deal with in their daily work, such as anthropogenic disturbance [46–48].

Furthermore, the formal goals of PA management, as indicated in the legal documents, when establishing National Parks, often include cultural services, like education, protection of cultural heritage, and recreation. For example, the regulation on the protection of Hardangervidda specifies that the aim of the park is to protect both the ecosystem itself and cultural services, including hunting, recreation and education (Regulation 4839/1981). Due to these formal aims and regular management of tourism activities in their PA, PA managers could be led to emphasise both anthropogenic threats and cultural ES [49].

The observed differences in views may also be an effect of the more in-depth and theoretical view of scientists on ecosystems, and the more general and practical view of managers [40]. The scientists may have a more detailed theoretical understanding of what is underpinning the ES in a PA, whereas the managers need to keep a broad overview of all processes and deal with the practical implementation, including societal aspects. For example, considering the ES that are provided by trees and undergrowth, the type of tree is of lesser importance as long as the ES such as carbon sequestration, flood mitigation, or erosion control themselves are sustained. Similarly, in the debate on the role of biodiversity, some studies argue that species traits are more important to the functioning of an ecosystem than the diversity itself [50–52]. Whereas detailed information may be superfluous for managers, the scientists require detailed knowledge to understand and model the system [11].

In addition, the higher variation in the perception of important variables among scientists than among managers (Table 3) may be caused by the same process, since the scientists are inclined to have a more detailed theoretical understanding of the system, therefore being able to come up with a wider variety of terms than the PA managers.

Of note here is that among stakeholders interested in the ES of a PA such as farmers or fishermen, the perception of ES may even be influenced by the scale and duration (in decades) that a PA has been managed and under protection [53–55]. The (duration of the) communication between PA managers and these stakeholders, and the creation of awareness and understanding, may increase the appreciation of the benefits of the management installed in a PA and the ES delivered by the PA. Similar factors may also influence the perception of ES and Threats in a PA by managers and scientists.

It has to be kept in mind that the concept of ES is highly anthropogenic [56], and therefore it is easy to forget about the structures and functions that underlie these services if one is not forced to do so. Nevertheless, for a full understanding of the functioning and potentials of a PA, it is advisable to account for the entire range of ecosystem elements when considering the complete flow from ecosystem structures and functions to ecosystem services and benefits, including the threats, and not to focus solely on the outcomes of a few elements in the system.

## Conclusion

Scientists and managers of PAs differ markedly in their view on the importance of various major ecosystem services and threats. Managers emphasised the anthropogenic (socio-economic and cultural) variables, and scientists underlined the importance of abiotic and biotic variables. Obviously, the perception of problems and challenges is biased by day-to-day business and workload. Therefore, it is advisable that in cooperation between scientists and managers, the social and economic factors, including the requirements and pressures of ecosystem services beneficiaries and practitioners, need to be linked more closely to the progress in natural sciences, including the abiotic and biotic processes underlying ecosystem functions and services and changes therein. Intensified and harmonised communication across disciplinary and professional boundaries is needed to improve ES oriented management strategies in existing PAs. This is also crucial when networks of PAs need to be adapted or when new PAs are installed. A more overarching approach will enable a more successful and realistic assessment of management strategies and policy options for current and novel PAs.

## Supporting information

S1 Table Example of the survey sent to, and answers from, the scientists working on protected areas. (PDF)

S2 Table Harmonisation tables for all variables. (S2a) ecosystem services, (S2b) threats, and the classification of the variables into variables of biotic, abiotic or socio-economic (anthropogenic) nature, grey cells are variables indicated by PA managers.
(PDF)

S3 Table Example of the survey sent to PA managers. (PDF)

S4 Table List of ecosystem types. Indicated for the transitional waters (TW) and the mountainous (MO) protected areas.
(PDF)

S5 Table List of mistakes made in the surveys and the ways used to correct them. Categories are Ecosystem Services (ES), Threats (Thr), and Ecosystem Types (ETy). The variable which was originally indicated (“between quotation marks”) is followed by our Remark on it (unless it may have been renamed). For the Actions taken: Split means that the term is split into two or three new terms, Rename means that the original term was renamed (and with its new name entered into the harmonisation tables of S2 Table), Omitted means the term was not used in the analysis (and in case of duplications one of the two terms was omitted). In the column ‘Renamed in’, the new name for the variable used in the analysis is given.
(PDF)
