# Supplementary material for: Ecosystem services in European protected areas: Ambiguity in the views of scientists and managers?
Source: PLoS One. 2017 Nov 15;12(11):e0187143. doi: 10.1371/journal.pone.0187143 (PMC5687704; doi:10.1371/journal.pone.0187143)
Supplement: S1 Table — (PDF) [file pone.0187143.s001.pdf]

S1. Example of the survey sent to, and answers from, the scientists working on protected areas.

Responsible scientific researcher filling in the table: Sander Wijnhoven

	Name of Protected Area (type of protection): Western Scheldt & Saeftinghe (Natura2000)		
**Habitat / Ecosystem type**	**Ecosystem service**	**Ecosystem functions and structures**	**Major threat(s)**
Tidal flats	Feeding grounds for birds and fish	Primary and secondary production	Increasing hydrodynamics, Increasing elevation and steepening edges (deepening for shipping); Increasing wave-action (more and larger boats); Reduction of intertidal area; Invading species
	Resting places for birds and mammals	Undisturbed habitats	Disturbance by recreants and food-collectors
	Cultural: Aesthetic values	Habitat heterogeneity	Reduction of intertidal area
	Cutting Sea-aster	Secondary production	Over-exploitation
Salt marshes	Protection of coastline	Habitat heterogeneity	Storm surges; Increasing hydrodynamics (deepening for shipping)
	Charismatic species	Breeding grounds for birds (biodiversity)	Disturbance by recreants); Reduction of area salt marshes (deepening for shipping); aging of marshes (obstruction of succession)
	Mediation of wastes	Nutrient cycling	Reduction of area, change in species composition (spatial planning)
	Tourism and wilderness experience	Habitat heterogeneity and biodiversity	Disappearance appreciated plant species (by eutrophication)
High dynamic gulleys	Fishing	Secondary production	Overfishing; Disturbance foodweb by pollutants; Disturbance foodweb by increasing sediment loads upstream (deepening for shipping)
	Waterway for supertankers	Surface, currents, hydrodynamics	Cons and impacts becoming larger than the benefits
	Cooling water intake	Buffering capacity, hydrodynamics	Invading species (fouling)
Low dynamic shallow waters (e.g. subtidal flats and small gulleys)	Nursery area for shrimps and fish	Habitat heterogeneity	Overfishing; Increasing hydrodynamics (deepening for shipping); Reduction of low dynamic shallow water areas
	Shellfish fisheries	Secondary production	Overfishing; Increasing hydrodynamics (deepening for shipping); Increasing water turbidity; Reduction of low dynamic shallow water areas; Invading species
