# Supplementary material for: Ecosystem services in European protected areas: Ambiguity in the views of scientists and managers?
Source: PLoS One. 2017 Nov 15;12(11):e0187143. doi: 10.1371/journal.pone.0187143 (PMC5687704; doi:10.1371/journal.pone.0187143)
Supplement: S2 Table — (S2a) ecosystem services, (S2b) threats, and the classification of the variables into variables of biotic, abiotic or socio-economic (anthropogenic) nature, grey cells are variables indicated by PA managers. (PDF) [file pone.0187143.s002.pdf]

S2: Harmonisation tables for all variables. (S2a) ecosystem services and (S2b) threats, and the classification of the variables into variables of biotic, abiotic or socio-economic (anthropogenic) nature, grey cells are variables indicated by PA managers

S2a:

Harmonised variable	Former (original) indication	Classification
Aesthetic qualities	Aesthetic qualities	Socio-economic
Aesthetic qualities	Cultural heritage	Socio-economic
Animals of economic use	Animal Production	Biotic
Animals of economic use	Aquaculture	Biotic
Animals of economic use	Bait collection	Biotic
Animals of economic use	Beekeeping	Biotic
Animals of economic use	Capture fisheries	Biotic
Animals of economic use	Cattle grazing	Biotic
Animals of economic use	Collecting of bait	Biotic
Animals of economic use	Commercial fisheries	Biotic
Animals of economic use	Fishing	Biotic
Animals of economic use	Food provision	Biotic
Animals of economic use	Honey production	Biotic
Animals of economic use	Manual cockle fisheries	Biotic
Animals of economic use	Oyster culture	Biotic
Animals of economic use	Shellfish fisheries	Biotic
Animals of economic use	Wild foods	Biotic
Animals of economic use	Agriculture, meat	Biotic
Animals of economic use	Farmed sea food	Biotic
Animals of economic use	Fisheries	Biotic
Animals of economic use	Wild land meat	Biotic
Biodiversity conservation	Biodiversity Conservation	Biotic
Biodiversity conservation	Biodiversity protection	Biotic
Biodiversity conservation	Refuge for biodiversity	Biotic
Biodiversity conservation	Genetic resources	Biotic
Charismatic landscape	Aesthetic values	Abiotic
Charismatic landscape	Charismatic habitat	Abiotic
Charismatic landscape	Charismatic habitat and species	Abiotic
Charismatic landscape	Charismatic landscapes	Abiotic
Charismatic landscape	Cultural heritage	Abiotic
Charismatic landscape	Cultural landscape	Abiotic
Charismatic landscape	Iconic landscapes	Abiotic
Charismatic species	Charismatic reindeer	Biotic
Charismatic species	Charismatic species	Biotic
Charismatic species	Existence value (of cetaceans)	Biotic
Charismatic species	Presence of flagship species	Biotic
Climate regulation	Carbon sequestration	Abiotic
Climate regulation	Carbon Uptake	Abiotic
Climate regulation	Climate regulation	Abiotic
Climate regulation	Local Scale Climate Regulation	Abiotic
Climate regulation	Carbon sequestration and storage	Abiotic

Education and research	Education	Socio-economic
Education and research	Research	Socio-economic
Education and research	Scientific research	Socio-economic
Education and research	Education	Socio-economic
Education and research	Research	Socio-economic
Energy production	Energy production (e.g. hydropower, wind farms)	Socio-economic
Energy production	Geothermic water	Socio-economic
Fire Protection	Wildfire regulation	Biotic
Flood and coastal protection	Buffer for coastal erosion	Abiotic
Flood and coastal protection	Buffering floods	Abiotic
Flood and coastal protection	Coastal protection	Abiotic
Flood and coastal protection	Flood and erosion protection	Abiotic
Flood and coastal protection	Flood mitigation	Abiotic
Flood and coastal protection	Flood retention	Abiotic
Flood and coastal protection	Protection of coastline	Abiotic
Flood and coastal protection	Flood prevention	Abiotic
Food provision for animals	Fodder	Biotic
Food provision for animals	Food for birds	Biotic
Food provision for animals	Food for cattle	Biotic
Food provision for animals	Food for fish	Biotic
Food provision for animals	Grazing	Biotic
Food provision for animals	Sheep fodder	Biotic
Food provision for animals	Reed as raw material or fodder	Biotic
Food provision for humans	Food collection	Biotic
Habitat for feeding and breeding	Breeding places and shelter for birds	Abiotic
Habitat for feeding and breeding	Feeding and staging grounds for birds	Abiotic
Habitat for feeding and breeding	Feeding grounds for birds	Abiotic
Habitat for feeding and breeding	Feeding grounds for fish	Abiotic
Habitat for feeding and breeding	Fishing ground	Abiotic
Habitat for feeding and breeding	Migration corridor for fish	Abiotic
Habitat for feeding and breeding	Nursery area	Abiotic
Habitat for feeding and breeding	Nursery area for shrimp and fish	Abiotic
Habitat for feeding and breeding	Nutrition for cattle	Abiotic
Habitat for feeding and breeding	Rangeland for cattle	Abiotic
Habitat for feeding and breeding	Resting place for birds	Abiotic
Habitat for feeding and breeding	Resting place for mammals	Abiotic
Habitat for feeding and breeding	Resting places for birds	Abiotic
Habitat for feeding and breeding	Resting places for mammals	Abiotic
Habitat for feeding and breeding	Sanctuary for fish fry	Abiotic
Habitat for feeding and breeding	Spawning and nursery grounds for fish	Abiotic
Habitat for feeding and breeding	Water for aquaculture	Abiotic
Habitat for feeding and breeding	Lifecycle and habitat protection	Biotic
Habitat for feeding and breeding	Nursery area - supporting	Biotic
Hunting	Hunting	Socio-economic
Hunting	Selling licenses	Socio-economic
Hydrological regulation	Hydrological cycle and water flow maintenance	Abiotic

Hydrological regulation	Hydrology	Abiotic
Leisure activities	Birdwatching	Socio-economic
Leisure activities	Ecotourism	Socio-economic
Leisure activities	Recreation	Socio-economic
Leisure activities	Recreation and tourism	Socio-economic
Leisure activities	Recreational activities	Socio-economic
Leisure activities	Recreational diving	Socio-economic
Leisure activities	Recreational fishing and boating	Socio-economic
Leisure activities	Symbolic and Aesthetic values	Socio-economic
Leisure activities	Tourism	Socio-economic
Leisure activities	Recreation and tourism	Socio-economic
Materials of economic use	Amber extraction	Abiotic
Materials of economic use	Cooling water	Abiotic
Materials of economic use	Mining	Abiotic
Materials of economic use	Salt production	Abiotic
Materials of economic use	Amber extraction	Abiotic
Materials of economic use	Gitios extraction	Abiotic
Materials of economic use	Salt production	Abiotic
Plants of economic use	Agriculture	Biotic
Plants of economic use	Biomass (wood, food)	Biotic
Plants of economic use	Biomass extraction	Biotic
Plants of economic use	Building material	Biotic
Plants of economic use	Cork Production	Biotic
Plants of economic use	Fruit crops	Biotic
Plants of economic use	Fuel pellets	Biotic
Plants of economic use	Pine seed extraction	Biotic
Plants of economic use	Plant collection	Biotic
Plants of economic use	Thatching materials	Biotic
Plants of economic use	Timber	Biotic
Plants of economic use	Wild plants and their outputs	Biotic
Plants of economic use	Agriculture , grain	Biotic
Plants of economic use	Timber	Biotic
Plants of economic use	Wild non meat food products	Biotic
Pollination	Pollination	Biotic
Pollination	Pollination and seed dispersal	Biotic
Pollination	Pollination	Biotic
Prevention of erosion	Control of erosion	Abiotic
Prevention of erosion	Erosion regulation	Abiotic
Raw materials	Sand, gravel, shell extraction	Abiotic
Resilience	Resilience	Biotic
Sedimentological regulation	Land incrementation	Abiotic
Sedimentological regulation	Mainenance of soil	Abiotic
Sedimentological regulation	Maintenance of soil fertility	Abiotic
Sedimentological regulation	Soil formation	Abiotic
Sedimentological regulation	Soil protection	Abiotic
Sedimentological regulation	Erosion prevention (coastal or inland)	Abiotic

Spiritual significance	Spiritual significance	Socio-economic
Transport facilitation	Shipping lanes	Socio-economic
Transport facilitation	Waterway for shipping	Socio-economic
Waste and Toxicant mediation	Denitrification	Abiotic
Waste and Toxicant mediation	Dewatering of wastewater treatment sludge	Abiotic
Waste and Toxicant mediation	Mediation of wastes	Abiotic
Waste and Toxicant mediation	Nutrient Regulation	Abiotic
Waste and Toxicant mediation	Pollution trapping	Abiotic
Waste and Toxicant mediation	Toxicity regulation	Abiotic
Waste and Toxicant mediation	Water filtration	Abiotic
Waste and Toxicant mediation	Water purification	Abiotic
Waste and toxicant mediation	Nutrient retention	Abiotic
Waste and toxicant mediation	Pest and disease control	Abiotic
Water regulation	Fresh water	Abiotic
Water regulation	Water storage	Abiotic
Water regulation	Water supply	Abiotic
Water regulation	Fresh water	Abiotic
Water regulation	Water treatment	Abiotic

2b:

Harmonised variable	Former (original) indication	Classification
(Illegal) human activities	Conflicting activities	Anthropogenic
(Illegal) human activities	Illegal catches	Anthropogenic
(Illegal) human activities	illegal logging	Anthropogenic
(Illegal) human activities	Picking of plants	Anthropogenic
(Illegal) human activities	Poaching	Anthropogenic
(Illegal) human activities	Gas extraction	Anthropogenic
(Illegal) human activities	Hunting	Anthropogenic
Agriculture	Agriculture	Anthropogenic
Agriculture	Agriculture	Anthropogenic
Bad management	Inappropriate water management	Anthropogenic
Bad management	Negligent management	Anthropogenic
Change in land use	Abandonment	Anthropogenic
Change in land use	Abandonment of farming	Anthropogenic
Change in land use	Changes in land use	Anthropogenic
Change in land use	Decrease of crops	Anthropogenic
Change in land use	Depopulation	Anthropogenic
Change in land use	Development of tourist facilities	Anthropogenic
Change in land use	Extension port areas	Anthropogenic
Change in land use	Forest management around the park	Anthropogenic
Change in land use	Harbour Extension	Anthropogenic
Change in land use	Settlements	Anthropogenic
Change in land use	Soil tillage	Anthropogenic
Change in land use	Spatial planning	Anthropogenic
Change in land use	Urbanisation	Anthropogenic
Change in species	Aging of the wild stocks	Biotic
Change in species	Bush encroachment	Biotic

Change in species	Change of plant species composition	Biotic
Change in species	Changes in bird dispersal	Biotic
Change in species	Disappearing charismatic species	Biotic
Change in species	Extinction of species	Biotic
Change in species	Food competition with cultured species	Biotic
Change in species	Impact of bird colonies	Biotic
Change in species	Plant species composition	Biotic
Change in species	Prey decline	Biotic
Change in species	Species composition	Biotic
Change in species	Species loss	Biotic
Change in species	Species reduction	Biotic
Change in species	Storms	Biotic
Change in species	Succession	Biotic
Change in species	Successional stagnation	Biotic
Change in species	Sudden oak death	Biotic
Change in species	Invasive species	Biotic
Civil engineering	Increased number of dams	Anthropogenic
Climate change	Change in precipitation	Climate change
Climate change	Change in snow cover	Climate change
Climate change	Changes in snow cover	Climate change
Climate change	Climate change	Climate change
Climate change	Droughts	Climate change
Climate change	Less precipitation	Climate change
Climate change	Sea Level Rise	Climate change
Climate change	Severe drought	Climate change
Climate change	Temperature changes	Climate change
Climate change	Climate change	Anthropogenic
Diseases	Diseases	Biotic
Diseases	Forest pests	Biotic
Diseases	Forests pests	Biotic
Diseases	Pests	Biotic
Diseases	Pests and diseases	Biotic
Disturbance	Anthropogenic disturbance	Anthropogenic
Disturbance	Disturbance	Anthropogenic
Disturbance	Disturbance by humans	Anthropogenic
Disturbance	Human actions	Anthropogenic
Disturbance	Human disturbance	Anthropogenic
Disturbance	Off-road Vehicles	Anthropogenic
Disturbance	Transport	Anthropogenic
Encroachment	Heath and scrub encroachment	Biotic
Encroachment	Tree Encroachment	Biotic
Eutrophication	Eutrophication	Anthropogenic
Eutrophication	Hypertrophic conditions	Anthropogenic
Eutrophication	Nitrification	Abiotic
Eutrophication	Eutrophication	Anthropogenic
Exotic species	Alien species	Biotic

Exotic species	Exotic Species	Biotic
Exotic species	Invading species	Biotic
Exotic species	Invasive Species	Biotic
Fire	Forest fire	Abiotic
Fire	Forest fires	Abiotic
Fire	Uncontrolled burning	Abiotic
Fire	Wildfires	Abiotic
Fisheries	Bycatch in gill nets	Anthropogenic
Fisheries	Fisheries	Anthropogenic
Fisheries	Shellfish fisheries	Anthropogenic
Fisheries	Fishing	Anthropogenic
Habitat loss	Aging of marshes	Abiotic
Habitat loss	Forest decay	Biotic
Habitat loss	Fragmentation	Anthropogenic
Habitat loss	Habitat change	Abiotic
Habitat loss	Habitat loss	Abiotic
Habitat loss	Habitat reduction	Abiotic
Habitat loss	Reduction of area	Abiotic
Habitat loss	Reduction of intertidal area	Abiotic
Habitat loss	Reduction of salt marshes	Abiotic
Habitat loss	Urban development	Anthropogenic
Habitat loss	Isolation	Abiotic
Habitat loss	Landscape fragmentation	Abiotic
Harmfull Algae	Algal blooms	Biotic
Harmfull Algae	Toxic algae	Biotic
Hydrological changes	Decrease of sediment transport	Abiotic
Hydrological changes	Deepening shipping lanes	Anthropogenic
Hydrological changes	Dredging	Anthropogenic
Hydrological changes	Hydraulic	Anthropogenic
Hydrological changes	Hydraulic modification	Anthropogenic
Hydrological changes	Hydroperiod reduction	Anthropogenic
Hydrological changes	Increased turbidity	Abiotic
Hydrological changes	Increasing hydrodynamics	Anthropogenic
Hydrological changes	Increasing sediment loads	Abiotic
Hydrological changes	Increasing turbidity	Abiotic
Hydrological changes	Increasing wave action	Anthropogenic
Hydrological changes	Reduced tidal energy	Anthropogenic
Hydrological changes	Storm surges	Abiotic
Hydrological changes	Underground water extraction	Anthropogenic
Hydrological changes	Water management	Anthropogenic
Hydrological changes	Water quantity	Abiotic
Increased salinisation	Groundwater salinisation	Abiotic
Increased salinisation	Hypersaline conditions	Anthropogenic
Landscape disturbance	Gas exploitation	Anthropogenic
Landscape disturbance	Visual ruining of landscape	Anthropogenic
Overexploitation	Harvesting	Anthropogenic

Overexploitation	Intensive agriculture	Anthropogenic
Overexploitation	Intensive Grazing	Anthropogenic
Overexploitation	Negative impact becoming larger than profits	Anthropogenic
Overexploitation	Overexploitation	Anthropogenic
Overexploitation	Overfishing	Anthropogenic
Overexploitation	Overgrazing	Anthropogenic
Overexploitation	Over-tourism	Anthropogenic
Overexploitation	Too high boat density	Anthropogenic
Overexploitation	Forestry	Anthropogenic
Overexploitation	Other biological resource extraction	Anthropogenic
Pollution	Air pollution	Anthropogenic
Pollution	Atmospheric Pollution	Anthropogenic
Pollution	Increased pollution	Anthropogenic
Pollution	Pesticides	Anthropogenic
Pollution	Pollution	Anthropogenic
Pollution	Water pollution	Anthropogenic
Pollution	Pollution	Anthropogenic
Pollution	Sonar and sound pollution	Anthropogenic
Predation	Predation	Biotic
Sediment dynamics changes	Avalanches	Abiotic
Sediment dynamics changes	Embankments within wetlands	Anthropogenic
Sediment dynamics changes	Erosion	Abiotic
Sediment dynamics changes	Port dredging	Anthropogenic
Sediment dynamics changes	Sediment disturbance	Anthropogenic
Sediment dynamics changes	Siltation	Abiotic
Sediment dynamics changes	Soil loss	Abiotic
Tourism	Hiking impact	Anthropogenic
Tourism	Mountaineering, rock climbing, speleology	Anthropogenic
Tourism	Recreation	Anthropogenic
Tourism	Recreational activities	Anthropogenic
Tourism	Tourism	Anthropogenic
Tourism	Tourism	Anthropogenic
