# Supplementary material for: Ecosystem services in European protected areas: Ambiguity in the views of scientists and managers?
Source: PLoS One. 2017 Nov 15;12(11):e0187143. doi: 10.1371/journal.pone.0187143 (PMC5687704; doi:10.1371/journal.pone.0187143)
Supplement: S3 Table — (PDF) [file pone.0187143.s003.pdf]

S3. Example of the survey sent to PA managers

Filled in by PA managers of the Curonian lagoon and Nenumas Delta

	**How important are the following ecosystem services to the beneficiaries of the PA?** (relative to the other ecosystem services, on a scale from 1 (least important) to 5 (most important) 0 = not important or unknown)						
	Ecosystem service	0	1	2	3	4	5
Provisioning services	Agriculture, meat	X	☐	☐	☐	☐	☐
	Agriculture , grain	X	☐	☐	☐	☐	☐
	Fisheries	☐	☐	☐	☐	☐	X
	Farmed sea food	X	☐	☐	☐	☐	☐
	Genetic resources	X	☐	☐	☐	☐	☐
	Timber	☐	☐	☐	☐	X	☐
	Wild land meat	X	☐	☐	☐	☐	☐
	Wild non meat food products (e.g. berries, mushrooms, kelp)	☐	X	☐	☐	☐	☐
	Fresh water	X	☐	☐	☐	☐	☐
	Energy production (e.g. hydropower, wind farms)	X	☐	☐	☐	☐	☐
	Please fill in if others:						
	Amber extraction	☐	☐	☐	☐	☐	X
	Geothermic water	☐	☐	☐	☐	☐	X
		☐	☐	☐	☐	☐	☐
Regulating services	Carbon sequestration and storage	☐	☐	☐	X	☐	☐
	Erosion prevention (coastal or inland)	☐	X	☐	☐	☐	☐
	Lifecycle and habitat protection	☐	☐	☐	☐	X	☐
	Pollination	☐	☐	☐	☐	☐	X
	Pest and disease control	☐	☐	☐	☐	☐	X
	Water treatment	X	☐	☐	☐	☐	☐
	Flood prevention	X	☐	☐	☐	☐	☐
	Please fill in if others:						
		☐	☐	☐	☐	☐	☐
Cultural services	Spiritual significance	☐	☐	☐	☐	X	☐
	Recreation	☐	X	☐	☐	☐	☐
	Education	☐	☐	X	☐	☐	☐
	Aesthetic qualities	☐	X	☐	☐	☐	☐
	Research	☐	☐	X	☐	☐	☐
	Please fill in if others:						
		☐	☐	☐	☐	☐	☐
Other	*Please fill in if others:*						
		☐	☐	☐	☐	☐	☐
		☐	☐	☐	☐	☐	☐

What are the most damaging environmental pressures or threats to your PA?				
**Environmental pressures**	High pressure	Medium pressure	Low pressure	No pressure
Agriculture	☐	☐	X	☐
Forestry	☐	X	☐	☐
Climate change	☐	☐	X	☐
Invasive species	☐	X	☐	☐
Eutrophication	X	☐	☐	☐
Tourism	X	☐	☐	☐
Pollution	☐	☐	X	☐
Hunting	☐	☐	☐	X

Fishing	☐	X	☐	☐
Other biological resource extraction (e.g. shells, berries)	☐	☐	X	☐
Transport	X	☐	☐	☐
Landscape fragmentation	☐	X	☐	☐
Please fill in if others:				
Sonar and sound pollution	☐	☐	☐	X
	☐	☐	☐	☐
