# Supplementary material for: Ecosystem services in European protected areas: Ambiguity in the views of scientists and managers?
Source: PLoS One. 2017 Nov 15;12(11):e0187143. doi: 10.1371/journal.pone.0187143 (PMC5687704; doi:10.1371/journal.pone.0187143)
Supplement: S4 Table — Indicated for the transitional waters (TW) and the mountainous (MO) protected areas. (PDF) [file pone.0187143.s004.pdf]

S4. List of Ecosystem Types. Indicated for the transitional waters (TW) and the mountainous (MO) protected areas.

**Ecosystem Type**	**Transitional Waters / Mountainous**
Aeolic sands with juniper forest and playa lakes	TW
Alpine and subalpine meadows	MO
Alpine Prairies	MO
Altitudinal transects from the Montane to the Alpine belt	MO
Coastal and marine ecosystems	TW
Coastal dunes and sea shore	TW
Coniferous and mixed mountain forests	MO
Cupressus Forests	MO
Freshwater and brackish marshes with emergent vegetation	TW
Freshwater ecosystems	TW
Fruit tree crops	MO
Grass lands	MO
Heath and Scrub	MO
High altitude Alpine Lakes	MO
High dynamic gulleys	TW
High mountain grasslands and shrub lands	MO
Lagoon fringe reed beds	TW
Lagoons	TW
Lichen fields	MO
Low dynamic shallow waters	TW
Mediterranean annual rich dry grassland	TW
Mediterranean shrub land with cork oak forest	TW
Mid mountain shrub lands	MO
Montado	MO
Montane Spruce-Fir-Beech forest	MO
Mountain lakes and surrounding meadows	MO
Native Deciduous Forest	MO
Natural forests	MO
Olea and Ceratonia forests	MO
Open Lagoon	TW
Permanent Grassland	MO
Pine forests	MO
Pine plantations	MO
Quercus forests	MO
River	TW
Rocks and screes	MO
Rocky Watersheds	MO
Salt marshes	TW
Seagrass Meadows	TW

Seasonal freshwater marshland	TW
Shrub lands	MO
Tidal Flats	TW
Wetlands	TW
