# Supplementary material for: Ecosystem services in European protected areas: Ambiguity in the views of scientists and managers?
Source: PLoS One. 2017 Nov 15;12(11):e0187143. doi: 10.1371/journal.pone.0187143 (PMC5687704; doi:10.1371/journal.pone.0187143)
Supplement: S5 Table — Categories are Ecosystem Services (ES), Threats (Thr), and Ecosystem Types (ETy). The variable which was originally indicated (“between quotation marks”) is followed by our Remark on it (unless it may have been renamed). For the Actions taken: Split means that the term is split into two or three new terms, Rename means that the original term was renamed (and with its new name entered into the harmonisation tables of S2 Table), Omitted means the term was not used in the analysis (and in case of duplications one of the two terms was omitted). In the column ‘Renamed in’, the new name for the variable used in the analysis is given. (PDF) [file pone.0187143.s005.pdf]

S5. List of mistakes made in the surveys and the ways used to correct them. Categories are Ecosystem Services (ES), Ecosystem Functions and Structures (EF), Threats (Thr), and Ecosystem Types (ETy). The variable which was originally indicated ("between quotation marks") is followed by our Remark on it (unless it may have been renamed). For the Actions taken: Split means that the term is split into two or three new terms, Rename means that the original term was renamed (and with its new name entered into the harmonization tables of S2), Omitted means the term was not used in the analysis (and in case of duplications one of the two terms was omitted). In the column 'Renamed in', the new name for the variable used in the analysis is given.

Area	***Category***	***"Original variable" and Remark***	***Action***	***Renamed in***
Camargue	ES	"Flood retention" is no service, but a function (buffering is the service)	Rename	Buffering floods
Camargue	ES	"Waterfowl hunting, fishing, cattle" are separate services	Split	
Camargue	EF	"Climate change attenuation, Sea level rise attenuation" have (as EF) no clear relation with the ES "sunbathing and swimming" nor with the Thr "destruction due to massive touristic frequentation"	Rename	Climate regulation
Camargue	EF	"Water epuration" (F: Epurification) is a service and not a function (nutrient cycling would have been better)	Rename	Element cycling
Curonian lagoon	ES	"Nutrient and toxic substance removal" are not the same. Therefore, split into nutrient control and toxicity control	Split	
Curonian lagoon	ES	Denitrification is not a service but a function	Rename	Waste and Toxicant mediation
Danube	EF	"Biological productivity" is not specific enough	Rename	Primary production
Danube	EF	"Landscape opportunity" is not a clear function nor structure	Rename	Charismatic landscapes
Doñana	Thr	"Phytophtora infestation" is too specific	Rename	Diseases
Doñana	Thr	"None" is not a useful term in the threats section	Omitted	
Eastern Scheldt	EF	"Breeding grounds for birds" is a service not a function	Rename	Habitat suitability
Eastern Scheldt	EF	"Salt water" is not a function, nor a service	Rename	Habitat suitability
Gran Paradiso	ES	"Cultural ecosystem services" is an indistinct, too much overarching, term	Omitted	
Gran Paradiso	EF / Thr	"Tree encroachment" is duplicated as function and threat; it is a threat	Omitted as EF	
Hardangervidda	ETy	"Reindeer Lichens Interaction"; an interaction is not an Ecosystem Type	Rename	Lichen fields

Hardangervidda	ETy	“Sheep and Browsing-grassing resources interaction”; an interaction is not an Ecosystem Type	Rename	Grass lands
Hardangervidda	ETy	“Grouse and shrub structure interaction”; an interaction is not an Ecosystem Type	Rename	Shrub lands
High Tatra	EF	“Water supply” is a service and not a function	Rename	Water regulation
High Tatra	ES / EF	“Climate regulation” is indicated as both service and function; function is renamed	Rename	Change of Microclimate
High Tatra	Thr	“B02.06” is a specification of B02	Omitted	
High Tatra	EF	“Genetic resources” and “Pharmacological resources” are not functions but services	Rename	“Gene pool” and “Primary production”
High Tatra	ES / EF	“2.3.1.1 - Pollination” is indicated as service and function; as function renamed	Rename	Population dynamics
High Tatra	ES	“3.2.2.1 Other cultural outputs – Existence” is an indistinct term	Rename	Charismatic habitat and species
High Tatra	EF	“Landscape opportunity” is a service not a function	Rename	Landscape
High Tatra	Thr	“Dispersed habitation” and “Urbanisation”, are merely duplications	Omitted	
High Tatra	ES	“3.1.1.1 Physical and intellectual interactions with biota, ecosystems, and landscapes” is too indistinct	Rename	Tourism
High Tatra	ES	“3.2.2.1 Other cultural outputs – Existence” is too indistinct	Rename	Tourism
High Tatra	ES / EF	“2.3.3.1 - Soil formation” is indicated as a function as well as a service; as service renamed	Rename	Sedimentological regulation
Oros Idi	EF	“Olive oil production” is a service, not a function	Rename	Primary production
Samaria	EF	“Biodiversity” and “Sea scape formation” are different functions	Split	
Samaria/Oros Idi	EF	“Habitat provision” is not a function nor structure	Rename	Habitat
Samaria/Oros Idi	EF	“Pollination” is not a function for beekeeping but a result of beekeeping	Rename	Population dynamics
Samaria/Oros Idi	EF	Water treatment is not a function but a service	Rename	Hydrodynamics
Sierra Nevada	ES / EF	“Hydrological cycle” and ”Water supply” are switched as service and function	Rename	“Hydrological regulation” and “Hydrodynamics”, resp.

Sierra Nevada	ES / EF	“Pollination” is indicated as service and function; renamed for EF	Rename	Population dynamics
Sierra Nevada	EF	“Evapotranspiration” is merely a duplication of ”Evaporation”	Omitted	
Sierra Nevada	EF	“Water supply” is not a function and merely a duplication of “Water regulation”	Omitted	
Western Scheldt	EF	“Secondary production” to obtain plants as Sea-aster should have been primary production	Rename	Primary production
Western Scheldt	EF	“Raw materials” is not a function to obtain sand and gravel (but the service itself)	Rename	Habitat suitability
